# Health system readiness for digital point-of-care diagnostics for febrile illness across Africa: A realist review

**DOI:** 10.1371/journal.pdig.0001820

**Published:** 2026-11-06

**Authors:** Shola Molemodile Dele-Olowu, Mamadu Baldeh, Flavia Bawa, Leonard Baatiema, Frances Baaba da-Costa Vroom, Luc de Witte, Emilia Udofia, Alfred Edwin Yawson, Julie Balen, NIHR Global Health Research Group on Digital Diagnostics for African Health Systems

**Affiliations:** 1 School of Public Health, University of Ghana, Accra, Ghana; 2 The Medical Research Council Unit, The Gambia, at the London School of Hygiene & Tropical Medicine, Banjul, The Gambia; 3 West African Centre for Cell Biology, University of Ghana, Accra, Ghana; 4 The Hague University of Applied Sciences, The Hague, The Netherlands; 5 Department of Community Health, University of Ghana Medical School, College of Health Sciences, University of Ghana, Accra, Ghana; 6 School of Nursing, Midwifery, Allied and Public Health, Canterbury Christ Church University, Canterbury, United Kingdom; Peking University First Hospital, CHINA

## Abstract

Digital point-of-care (POC) diagnostics, including connected and molecular platforms, have the potential to strengthen clinical decision-making and disease surveillance, particularly for febrile illness across African health systems. Yet context-relevant evidence on health system readiness and the institutional conditions shaping their integration and sustainability is limited. Following RAMESES standards, we conducted a realist review to examine how, why, and under what circumstances digital POC diagnostics for febrile illness are integrated into African health systems. We systematically searched PubMed and CINAHL (2005–2025) and supplemented these with purposive searches of the grey literature. Qualitative and mixed-methods studies addressing implementation, integration, or readiness were included. Data were synthesized using context–mechanism–outcome (CMO) configurations to develop and refine the program theory. Seventeen studies from multiple African countries were included. Readiness for integration was explained by five interacting system levers: (1) usability and workflow compatibility; (2) workforce competence, trust, and confidence; (3) reliable infrastructure and connectivity; (4) governance, policy alignment, and financing; and (5) data systems that generate actionable value. When these conditions aligned, mechanisms such as perceived usefulness, confidence, institutional legitimacy, and responsiveness were activated, leading to sustained use, improved diagnostic pathways, and enhanced surveillance. Conversely, weaknesses, particularly in infrastructure, procurement, or data-use capacity, triggered frustration, distrust, and discontinuation. The integration of digital POC diagnostics into African health systems depends more on the health system’s readiness to promote trust, continuity, and data-driven action than on the performance of the tests themselves. A realist, system-focused approach is required to enable context-specific implementation and prevent the reinforcement of pre-existing inequities in digital health adoption.

## Author summary

Limited access to reliable diagnostics remains a major challenge across many African health systems, where febrile illnesses are often treated based on symptoms instead of confirmed diagnoses. Digital point-of-care (POC) diagnostics—portable devices that quickly test patients and send results electronically—are becoming more popular as tools to improve clinical decision-making and disease monitoring. However, for these technologies to work effectively, healthcare facilities need to be ready to integrate them into routine care. In this study, we conducted a realist review of the literature to understand the methods, rationale, and conditions under which digital POC diagnostics are integrated into African health systems. Through the synthesis of evidence from 17 studies across multiple countries, we identified five key factors influencing readiness: device usability, workforce competence and reliability, infrastructure dependability, the effectiveness of governance and financing, and the capacity of data systems to provide relevant information. The findings suggest that digital diagnostics are most effective when health systems foster trust, continuity, and data-driven decision-making, offering practical guidance for policymakers and implementers of digital health technologies.

## Introduction

Diagnostics are a core component of health systems, serving as a cross-cutting element within the World Health Organization’s (WHO) health system building blocks. They inform everyday clinical care and service-delivery decisions while also supporting broader population-level functions, such as disease surveillance, outbreak detection, and health system planning, financing, and governance [1–4]. In contexts where diagnostics are limited, fragmented, or poorly integrated into national health systems, patients risk receiving delayed or inappropriate treatment; health services face challenges in efficiently allocating resources and health authorities lack timely information to guide policymaking, system responsiveness, and preparedness efforts [5–7]. Across many African countries, diagnostic systems have developed in diverse and uneven ways, reflecting historical investment patterns, variable laboratory capacity, fragmented data and information flows, and differing regulatory and financing arrangements, resulting in persistent gaps in access to diagnostics [8–10].

Febrile illnesses are among the most common reasons for healthcare-seeking in many African countries and can have a wide range of causes, including malaria, typhoid, dengue, and other viral or bacterial infections [7,11–14]. Historically, limited access to diagnostics has led to symptom-based or presumptive treatment of fever rather than a confirmed diagnosis, contributing to potential misdiagnoses, overuse of antimicrobials, and poor patient outcomes [5,6,15]. Although serological assays and rapid diagnostic tests (RDTs) for malaria have brought significant improvements, their use remains variable across settings [16], and they provide limited guidance for patients with mixed infections or non-malarial febrile illness, in whom they may yield negative results. Although a growing body of evidence addresses digital health implementation and adoption of point-of-care (POC) diagnostics within African health systems, most of these are focused on disease programmes, specific technologies or implementation barriers with less emphasis on the broader health system readiness for integration. At the same time, febrile illness remains the most common cause of health seeking and a major driver of antibiotic misuse across Africa, and is rarely viewed from a health systems readiness lens [13,17,18].

Moreover, while these initiatives expanded diagnostic availability, they also highlighted substantial variation in how new diagnostics were introduced and scaled up (or not) across different health system contexts. Febrile illness thus exposes important system-level factors that extend beyond the performance of individual diagnostic tests.

Substantial efforts have been made to strengthen diagnostic systems across Africa, including regional collaborations to enhance laboratory capacity and advocacy for standards such as the “REASSURED” and “RESTASSURED” criteria to guide the design and development of new point-of-care (POC) diagnostics [18–22]. More recently, digital molecular technologies, such as “lab-on-chip” devices that combine molecular detection with connectivity, portability, and rapid turnaround times, have been proposed as novel approaches to bridge diagnostic gaps at the POC, especially in settings where laboratory infrastructure is limited or inaccessible [18,22]. However, wide variations in health and digital infrastructure, regulatory capacity, organisational maturity, workforce preparedness and financing arrangements mean that digital diagnostics are developed, introduced, governed, scaled up and sustained (or not) in highly heterogeneous contexts. Digital diagnostics are, therefore, not simply technical solutions but instead represent system-level interventions within diverse and complex environments, with important implications for health system readiness to integrate and sustain their use.

Evidence on African health systems’ readiness for new digital POC diagnostics is limited, and why similar diagnostic technologies generate different outcomes across settings remains poorly understood. Existing studies often focus on diagnostic performance, offering limited insight into how institutional arrangements, organisational conditions and stakeholder practices interact to shape readiness for diagnostic innovation. Where readiness is assessed, it is frequently treated as a static attribute, measured through indicators or checklists, rather than as a dynamic condition involving interactions between technologies, institutions and people. This limits policymakers’ and implementers’ ability to anticipate where, how, and why digital diagnostics are likely to succeed or fail, and to tailor strategies accordingly. Without contextual adaptations that support readiness, digital diagnostic innovations risk reinforcing existing inequalities and bottlenecks, rather than strengthening equitable diagnostic systems across African countries.

Addressing this gap requires moving beyond a traditional focus on diagnostic accuracy or effectiveness to account for complexity, interaction and contextual variation in health systems readiness. A *realist* approach conceptualises readiness not as a fixed capacity, but as an emergent outcome generated through mechanisms that are activated, or suppressed, by specific contexts. Such approaches have been applied across different areas of health policy, health systems and digital health research, demonstrating the value of theory-driven synthesis in unpacking complexity and informing context-sensitive decision-making [23–26]. This realist review uses context-mechanism-outcome (CMO) configurations to synthesise evidence on health system readiness for integrating digital POC diagnostics for febrile illness in settings across Africa, answering the research question:

*What contextual factors, mechanisms, and stakeholder responses explain how, why, and under what circumstances health systems in African countries integrate (or fail to), digital point-of-care diagnostics for febrile illnesses? Additionally, how do these factors influence health system readiness for such integration?*

## Methods

### Design

This realist review does not seek to assess implementation success or effectiveness, but to explain how health system readiness for digital POC diagnostics is generated or undermined through interacting contextual conditions and mechanisms. It was conducted and reported in accordance with the **ReA**list **ME**ta-narrative **S**ynthesis: **E**volving **S**tandards (RAMESES) publication standard [24], through iterative stages, namely: development of an initial program theory (IPTs) from scoping literature and stakeholder input; systematic and purposive searching of the literature to test and refine IPTs; data extraction from included studies and synthesis into refined CMO configurations and validation of emerging program theories. In compliance with the RAMESES publication standards, the review provides appropriate methodological guidance for realist reviews that explain the mechanisms and contextual factors influencing effectiveness of interventions, rather than strictly following PRISMA guidelines aimed primarily at synthesizing evidence in traditional systematic reviews and meta-analyses.

#### Phase 1: Developing an initial program theory.

Developing an IPT involved numerous team discussions and an initial scoping of the existing literature on digital POC testing or interventions, health technology integration, and implementation in low- and middle-income countries, to inform a preliminary idea. Scoping was conducted via a purposive Google Scholar literature search to gain a clear understanding. The review team, which included public health professionals, medical practitioners, and researchers, discussed and brainstormed initial ideas. An initial program theory was drafted based on the existing literature:

*“Readiness to integrate digital POC diagnostics for management of febrile illness (Outcome) is shaped by the responses and practices that are triggered when stakeholders interact with diagnostic technologies that are embedded in, and interact with, institutions (Mechanism), across diverse African health systems (Context).”*

This initial theory posits that readiness for a diagnostic technology is shaped by how actors interact with it within a specific institutional setting, rather than solely by its design. It seeks to explain the underlying social dynamics behind integration and implementation outcomes. This provides a pathway to developing digital POC tools in health system settings across African countries and designing contextually appropriate implementation strategies to improve adoption.

#### Phase 2: Systematic and purposive searching of the literature to refine the IPTs.

Search strategy. A systematic but pragmatic search strategy was used to identify relevant documents and refine the initial program theories. The search combined traditional systematic review methods with purposive searching. The PICO framework (Population, Intervention, Comparison, Outcome) was used to develop the review’s search strategy, as shown in Table 1.

**Table 1:** Framework for the review using PICO without a comparator.

Criteria	Inclusion	Exclusion
**Population**	Health systems, institutions and stakeholders in African countries	outside Africa
**Intervention**	Introduction, implementation and/or integration of digital POC diagnostic tools (including lab-on-chip) for febrile illnesses	Non-digital diagnostics or diagnostics unrelated to febrile diseases
**Outcomes**	Health system readiness for integration, i.e., capacity to provide diagnostic services according to defined standards such as guidelines, trained staff, equipment, digital infrastructure, commodities; systems to support quality and safety, user competencies, policy environment, stakeholder perceptions, integration factors or implementation outcomes	Studies not reporting relevant health system outcomes
**Study Design**	Qualitative, mixed methods, implementation science studies, policy analysis, grey literature	Purely quantitative studies and letters with no primary data
**Language**	English	Non-English without translation
**Timeframe**	2005–2025 (reflecting two decades of evolution of digital health in LMICs)	Studies published before 2005

The PICO framework frequently guides the selection of suitable search terms. Peer-reviewed literature was assessed using MeSH terms and keywords, including “digital diagnostics,” “febrile illness,” “point-of-care diagnostics,” “Africa,” “health system integration,” and “readiness.”

The search was restricted to English-language studies published between 2005 and 2025 and was conducted through an iterative process of piloting and refining the search terms to ensure that all relevant studies were captured. The systematic search was conducted using PubMed and CINAHL. PubMed is a free database of biomedical literature maintained by the National Institutes of Health (NIH), while CINAHL is a subscription-based, comprehensive database of journals and other materials related to nursing and allied health professions. It was accessed through the University of Ghana Library on August 21st, 2025. Wildcard symbols were used to incorporate all variations and spellings of the terms. All search terms were combined with the Boolean operator ‘OR,’ and the different concepts were linked with the operator ‘AND’. Inclusion and exclusion criteria are also outlined in Table 1. The full list of search terms is in S1 Text.

The purposive search involved grey literature searches using Google Scholar to identify relevant literature and citation chasing of the included literature. A subject matter expert in the field was also contacted for recommendations on additional literature.

#### Phase 3: Appraising and synthesising evidence.

Evidence appraisal was conducted using Covidence systematic review software. A total of 2,093 studies were identified, of which 601 were duplicates (see Fig 1 for PRISMA flow diagram).

**Fig 1:**
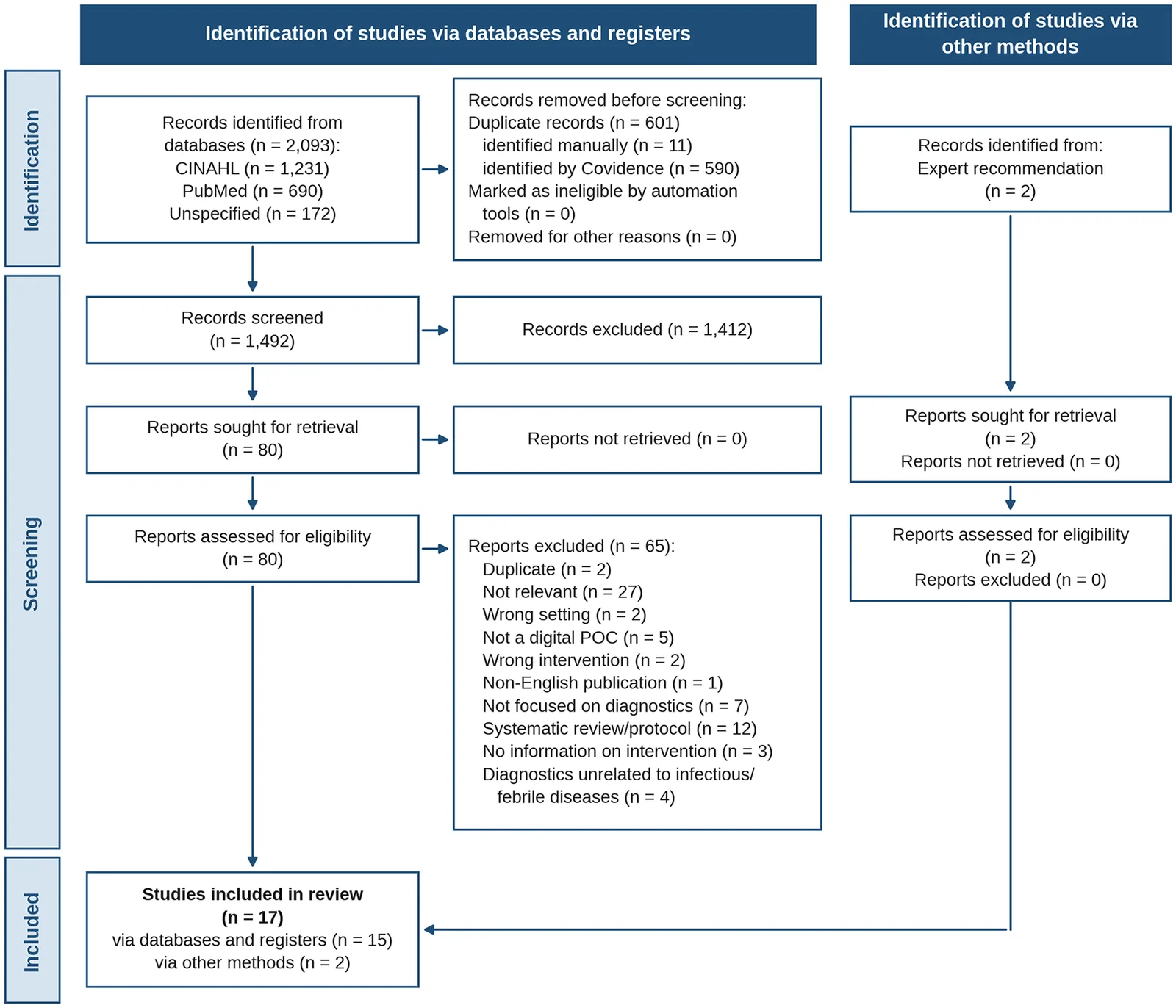
PRISMA 2020 flow diagram of selected studies [27].

POC, point-of-care. Two independent reviewers (SMDO & MB) screened titles and abstracts to ensure accurate identification of relevant studies. Studies did not need to include all components (e.g., digital point-of-care, febrile illness) because the initial scoping indicated that a narrow inclusion criterion would exclude important concepts, such as the application of digital interventions in point-of-care settings. However, articles that described an intervention but did not explain its underlying mechanisms were excluded. The reviewers resolved discrepancies and conflicts through consensus or consultation with a third reviewer (FB). This included verifying the article’s relevance and the extent to which it contained data that would inform theory development. This screening process reduced the pool to 80 relevant studies, for which full texts were obtained and reviewed. During full-text screening, 65 studies were excluded for poor data quality or irrelevant outcomes, leaving 15 for inclusion in the final review. To complement the systematic search strategy, two [2] additional studies were purposively identified based on recommendations from a subject-matter expert.

Data extraction was conducted using a structured matrix to systematically capture and document information from the included studies, including study title, publication year, study design, participant size and characteristics and study inclusion and exclusion criteria. In addition, data were extracted specifically to inform the development of context-mechanism-outcome configurations. Contexts were defined as features of the health system or implementation setting in which the intervention was introduced and experienced, including infrastructure, policy and regulatory environments, organisational routines and stakeholder relationships. Mechanisms were defined as the underlying processes – such as the reasoning, responses, or reactions of individuals or organisations – triggered by the intervention and leading to the observed outcomes within contexts. Outcomes captured the observed effects of interventions, including both intended and unintended consequences related to the integration, use, non-use, or abandonment of digital POC tests.

The extracted CMOs were iteratively grouped and analysed across studies to identify recurring patterns and themes, which were used to refine the initial programme theories through realist reasoning. A CMO configuration table was used to organize the data and to support potential explanations of how, why, to what degree, and in what ways digital diagnostic interventions were successfully integrated or not. The data extraction matrix and CMO synthesis and categorization are provided in S1 File and S2 File respectively.

#### Phase 4: Validation of emerging program theories.

Following recommended realist review methods, the final step was to validate the refined program theories against the literature and the implementation evidence identified in the review. This was done through an iterative process of reciprocal comparison: comparing candidate theories with specific research in a series of steps to assess how well they explained the data. If differences were found, the theories were revised or expanded. This step ensured that the improved context-mechanism-outcome (CMO) configurations were supported by real-world data and could explain differences across settings. Particular attention was paid to whether mechanisms identified in one context could be applied in another, thereby strengthening the transferability of findings.

## Results

### Study selection and characteristics

A total of seventeen [17] studies met the inclusion criteria and were analysed as part of this realist review (see Table 2 for the full list of studies).

**Table 2:** Characteristics of articles examined for the study.

Reference	Title	Country (or region)	Diagnostic Technology / Digital Tool	Aim	Study Design	Population
**Steenkamp et al. (2025)**	Digital health readiness – insights from healthcare leaders in operational management: a cross-sectional survey	South Africa	E-Ready 2.0 digital health readiness assessment scale (no specific POC diagnostic tool evaluated)	To assess perceived digital health readiness of healthcare leaders in public hospitals using the E-Ready 2.0 scale	Cross-sectional study	Healthcare leaders (nurses and medical doctors) in operational management in 11 public hospitals, Western Cape
**Bimerew (2024)**	Barriers and Enablers of Nurses’ Adoption of Digital Health Technology to Facilitate Healthcare Delivery in Resource-Limited Settings	South Africa	General digital health technologies (platform unspecified; nurse adoption context)	To investigate barriers and enablers of nurses’ adoption of digital health technology	Cross-sectional study	Nurses in 23 randomly selected primary healthcare centres in the Cape Town metropole, Western Cape
**Digital Diagnostics for Africa Network (2022)**	The potential of digital molecular diagnostics for infectious diseases in sub-Saharan Africa	Sub-Saharan Africa	Digital molecular diagnostics; lab-on-chip POC platforms for infectious disease detection	To review the need for improved diagnostics and disease surveillance in SSA and how digital diagnostics could meet this need	Narrative review	Not applicable (narrative review)
**Boniface et al. (2024)**	Xpert HIV-1 Qual point-of-care testing for HIV early infant diagnosis in Tanzania: experiences and perceptions of healthcare workers	Tanzania	GeneXpert Xpert HIV-1 Qual — molecular POC test for HIV early infant diagnosis (EID)	To assess the feasibility of nurse-based POC HIV early infant diagnosis in five facilities	Cross-sectional study	28 healthcare workers in Mbeya Region including nurses, clinical officers, laboratory scientists and technicians
**VanSteelandt et al. (2017)**	Operational evaluation of rapid diagnostic testing for Ebola Virus Disease in Guinean laboratories	Guinea	Ebola virus rapid diagnostic test (RDT)	To evaluate operational measures (acceptability, feasibility, quality assurance) during RDT implementation and assess concordance with PCR testing	Operational evaluation	Laboratory staff in high-risk areas of Guinea; patients suspected of Ebola infection
**Whittaker et al. (2011)**	What is the point of the point-of-care? A case study of user resistance to an e-health system	South Africa	POC e-health system (electronic patient records at point of care)	To investigate reasons behind nurses’ resistance to a POC e-health system and understand how cultural and work-related factors influence adoption	Case study	End-users including healthcare professionals, management, healthcare IT staff, and top management of the hospital
**Karemere et al. (2025)**	Evaluating the Implementation of Automated Malaria Rapid Diagnostic Test Readers in Health Facilities in the Democratic Republic of Congo	Democratic Republic of Congo	Automated malaria RDT reader (Deki Reader — digitally enabled device with digital data capture)	To assess the feasibility, process, challenges, and lessons learned from deploying automated malaria RDT readers across three provinces	Implementation study / operational field evaluation	Health workers and patients attending 144 health facilities in Haut Katanga, Kasai Central, and Sud Kivu provinces
**Tran Ngoc et al. (2018)**	Conclusions of the digital health hub of the Transform Africa Summit (2018): Strong government leadership and PPPs are key prerequisites for sustainable scale-up of digital health	Rwanda	Digital health platforms (policy and governance context; no specific POC diagnostic tool evaluated)	To summarise discussions and conclusions from the Digital Health Hub regarding strategies for sustainable scale-up of digital health in Africa	Conference proceedings	Government officials, private sector representatives, and health system experts from across Africa
**Swartz et al. (2021)**	Multiple pathways to scaling up and sustainability: an exploration of digital health solutions in South Africa	South Africa	Multiple digital health solutions (varied platforms including digital diagnostic and reporting systems)	To understand factors influencing the scaling and sustainability of digital health solutions	Qualitative research	Governmental and non-governmental stakeholders in health policy, implementation, technology, academia, and funding
**O'Brien et al. (2023)**	Strengths, Weaknesses, Opportunities, and Threats Analysis of the Use of Digital Health Technologies in Primary Health Care in Sub-Saharan Africa	Sub-Saharan Africa	Digital health technologies in primary care (general; varied platforms and tools)	To capture multidisciplinary experiences with digital health technologies to explore strengths, weaknesses, opportunities, and threats in SSA primary care	Qualitative research	Primary care professionals (clinical and non-clinical) with digital health technology experience in SSA PHC settings
**Moetlhoa et al. (2024)**	Barriers and enablers for implementation of digital-linked diagnostics models at point-of-care in South Africa: stakeholder engagement	South Africa	Digital-linked POC diagnostic models (multiple POC tests integrated with digital data capture and connectivity systems)	To identify barriers and enablers influencing the implementation of digital-linked POC diagnostic models in South Africa	Text and opinion (stakeholder engagement)	Researchers, academics, diagnostic test manufacturers, and key stakeholders in digital diagnostics
**Ibeneme et al. (2021)**	Data revolution, health status transformation and the role of artificial intelligence for health and pandemic preparedness in the African context	Africa	Artificial intelligence and digital health systems (general policy and governance context)	To discuss and propose solutions for responsible development and adoption of AI and digital innovations in Africa	Text and opinion	Policymakers, technical experts, academia, public and private sector partners
**Gous et al. (2018)**	The impact of digital technologies on point-of-care diagnostics in resource-limited settings	Not specified (global review)	Digital technologies for POC diagnostics (general review; covers connectivity, data capture, and reporting systems)	To review the potential impact and benefits of digitising POC diagnostics in resource-limited settings	Text and opinion (review)	Not applicable (review article)
**Ephraim et al. (2024)**	Application of medical artificial intelligence technology in sub-Saharan Africa: Prospects for medical laboratories	Sub-Saharan Africa	Artificial intelligence (AI) in medical laboratories (including AI-assisted diagnostic tools)	To explore prospects, challenges, and opportunities of medical AI technology in SSA laboratories and provide context-specific recommendations	Narrative review	Healthcare systems, medical laboratories, and stakeholders (policymakers, health professionals, patients) in SSA
**Aboye et al. (2024)**	Assessment of the Barriers and Enablers of mHealth Systems in Sub-Saharan Africa According to Perceptions of Patients, Physicians, and Health Care Executives in Ethiopia	Ethiopia	mHealth systems (mobile health applications for healthcare delivery)	To identify barriers and enablers for mHealth system use from perspectives of patients, physicians, and health care executives	Qualitative research	Patients, physicians, and health care executives (office directors, hospital directors, coordinators, ICT heads) attending chronic disease OPDs
**Ward et al. (2020)**	Usability and acceptability of an automated respiratory rate counter to assess children for symptoms of pneumonia: A cross-sectional study in Ethiopia	Ethiopia	ChARM automated respiratory rate counter (digital device for pneumonia screening in children)	To determine usability of the ChARM automated respiratory rate counter by health extension workers and its acceptability to workers and caregivers	Cross-sectional study	131 health extension workers conducting 262 sick child consultations after initial training and 337 at 2-month follow-up
**Bahati et al. (2025)**	Artificial intelligence (AI)-driven rapid diagnostic test interpretation in a Connected Diagnostics (ConnDx) system for dynamic malaria surveillance	Kenya	ConnDx AI-enabled malaria RDT system (mobile app + cloud infrastructure + AI image analysis for mRDT result interpretation and real-time surveillance)	To explore use of the ConnDx integrated digital platform for malaria surveillance across five health facilities	Cross-sectional study	3,620 mRDT tests across five health facilities in Kisumu County (May–December 2023); mixed age/sex distribution

Although we searched for studies published in the previous 20-year period (2005–25), all included studies were published between 2011 and 2025. The 17 studies were carried out across different African countries, with five (5) studies concentrated in South Africa [21,28–31]. Some studies were multi-country: three focused on sub-Saharan Africa, and two were Africa-wide. Most studies were conducted in primary health care facilities (4), in a combination of facility types (3), and 4 studies were unspecified, as they were either reviews or text opinions. Study designs varied, with qualitative studies and cross-sectional research being the most common (5 each), followed by opinion pieces (3) and narrative reviews (2), with one implementation study.

#### Nature of diagnostic technologies studied.

In terms of diagnostic focus, most studies examined general point-of-care (POC) testing and broader digital diagnostic programmes, including digital reporting systems. Only two studies explicitly addressed malaria rapid diagnostic tests [32,33], while a single study considered more advanced molecular or lab-on-chip technologies [18].

The context–mechanism–outcome (CMO) analysis highlighted five recurring configurations that explain how readiness to integrate digital diagnostics is produced, sustained or undermined in many African health systems. This is summarized in Fig 2.

**Fig 2:**
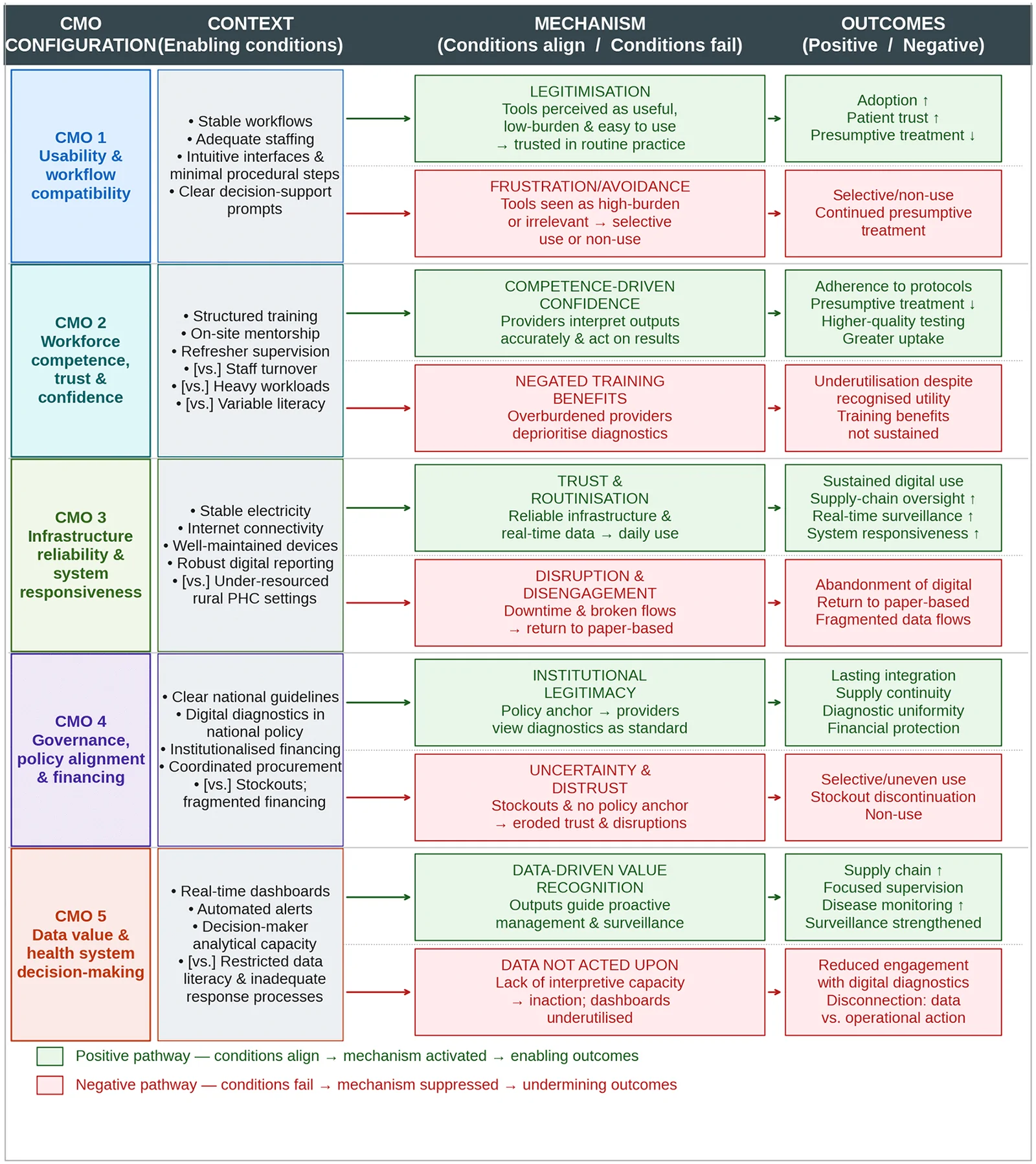
Health system conditions (levers) and mechanisms shaping readiness for integration of digital point-of-care diagnostics for febrile illness in Africa.

Fig 2 illustrates the refined program theory developed through this realist review, showing how key health system conditions (levers) across different contexts shape the activation of mechanisms that generate or undermine readiness to integrate digital POC diagnostics for febrile illness in African health systems.

#### CMO 1: Usability and clinical acceptability.

When digital tools are intuitive and fit existing workflows, healthcare providers perceive them as clinically useful and integrate them into routine care.

The most frequently cited characteristics influencing integration were usability and acceptability; eight studies reported that perceived ease of use, workflow compatibility, and clinical relevance and usefulness shaped uptake [17,28–34].

**Context.** Supportive contexts included stable workflows in health facilities, adequate staffing, and routine exposure to diagnostic tools. While some devices were designed with simple interfaces, minimal procedural steps, and clear decision-support prompts, enabling alignment with existing consultation routines, others added tasks or disrupted established workflows, increasing health workers’ cognitive and administrative burdens.

**Mechanism.** When digital POC diagnostics were aligned with routine workflows and supported clinical decision-making, health workers perceived them as clinically useful, easy to use, helpful, and low burden. These positive perceptions activated a mechanism of legitimisation, whereby diagnostic outputs were trusted, incorporated into routine practice and results communicated to patients with confidence, rather than treated as optional or peripheral tools or processes with limited clinical usefulness.

**Outcome.** As a result, when this mechanism was triggered, device adoption and use increased, patient trust in diagnostic processes improved, and reliance on presumptive treatment decreased. Conversely, when perceived clinical usefulness was low or processes were difficult to follow, users became frustrated and avoided the devices leading to selective use or non-use, despite technical accuracy.

#### CMO 2: Workforce competence, trust, and confidence.

When training and supervision accompany digital POC deployment, users develop competence and build confidence required to trust outputs and modify practice.

Seven [7] studies emphasised the role of training, capacity-building and supportive supervision in enhancing provider competence and confidence [17,28,29,33,35–37].

**Context.** Studies described the availability of structured training packages, on-site mentorship, or refresher supervision as enablers of integration. This is in the context of high staff turnover and heavy workloads typical in many PHC settings. Additionally, digital literacy and familiarity with digital tools varied across health facilities.

**Mechanism.** Training and supervision activated a competence-driven confidence mechanism, enabling providers to understand diagnostic processes and interpret diagnostic outputs accurately. This confidence reduced uncertainty and increased trust in the tool, thereby encouraging health workers to act on the results. For example, when paired with targeted training and supervision, healthcare providers in Kenya built trust in AI-enabled malaria rapid diagnostic tests (ConnDx) [37]. Additionally, three [3] studies highlighted the role of trust, both among providers and patients, as a mechanism linking perceived reliability with diagnostic uptake [17,28,35].

**Outcome.** Competence-based interventions encouraged adherence to testing protocols, reduced reliance on presumptive treatment, and built trust in digital diagnostic tools. Healthcare providers adhered more consistently to diagnostic testing protocols, reduced the use of presumptive treatment, and felt empowered to use digital outputs, resulting in higher-quality testing, improved accuracy, and greater uptake. However, in contexts of chronic understaffing, heavy workloads negated the benefits: training did not translate into sustained use. The same body of evidence showed that without addressing high workloads and staffing shortages, the benefits of training were limited, as overburdened providers often deprioritized digital diagnostics, despite recognizing their utility.

#### CMO 3: Infrastructure reliability and system responsiveness.

When digital infrastructure and connectivity are reliable, data flows generate feedback loops that reinforce use and strengthen diagnostic pathways.

The reliability of digital infrastructure was identified as a crucial contextual factor influencing the integration and sustained use of digital diagnostic tools in the studies examined. In environments with a steady electricity supply and internet connectivity, digital diagnostics were more frequently integrated into standard clinical and reporting workflows. In contrast, infrastructural fragility, particularly in inadequately funded rural public primary healthcare facilities, consistently limited acceptance and sustained use. Seven [7] studies documented how unreliable power supply, inadequate equipment, or poor internet access were contextual factors that undermined adoption across public, mostly rural and under-resourced primary health care centers [17,28,29,31,38–40]. These limitations caused device downtime and disrupted data flows, resulting in user frustration and, in some cases, the discontinuation of digital components and a return to paper-based or presumptive practices.

In contrast, eight [8] studies reported that robust data systems and digital reporting features were critical enablers of adoption and scale, providing actionable insights for clinical decision-making, supply-chain management, and disease surveillance [17,28–31,32,36,37]. These studies, conducted across a mix of public and private facilities at various levels of the health system and combining diagnostics with real-time feedback loops, enhanced health system responsiveness and improved stock management. In these contexts, reliable data flows supported actionable clinical insights, strengthened disease surveillance, and enabled more responsive supply-chain management, particularly for test kits and consumables.

**Mechanism.** Reliable infrastructure reduced users’ uncertainty and cognitive load by ensuring that diagnostic results, reporting systems, and feedback mechanisms functioned as intended. This reliability cultivated trust in the device and strengthened views of its utility, promoting regular usage and incorporation into daily practice.

As digital outputs became visible and actionable through automated notifications, dashboards, and inventory updates, users increasingly relied on real-time data to inform clinical and operational decisions. Over time, these feedback loops reinforced routinization, thereby facilitating the continued adoption and normalization of digital diagnostics within facility procedures.

**Outcome.** These findings demonstrate a distinct CMO configuration in which stable infrastructure and interoperable digital systems create enabling conditions that facilitate trust, confidence, and reliance on data, resulting in outcomes such as sustained usage, improved supply-chain oversight, and enhanced real-time surveillance. In contrast, infrastructure deficiencies create avenues for frustration and disengagement, leading to the abandonment of technology and a return to non-digital methods. This dual pattern underscores the importance of infrastructure preparedness for both the initial adoption and the long-term viability and scalability of digital diagnostic interventions within African health systems.

#### CMO 4: Governance, policy alignment, and financing.

When policies, guidelines, and financing streams recognise and institutionalise digital diagnostics, providers and managers perceive them as legitimate and sustainable.

Seven [7] studies highlighted the importance of clear national guidelines and policy inclusion to legitimise and sustain new diagnostic interventions [17,18,28,35–37,41].

Clear guidelines, particularly those defining where and how digital diagnostics should be used, minimized ambiguity for implementers and frontline providers by aligning new tools with existing diagnostic algorithms and clinical workflows. From a realist perspective, this policy anchoring was a crucial contextual factor that shaped stakeholders’ perceptions of the legitimacy and future of digital diagnostics within their healthcare systems.

Financing and procurement arrangements either strengthened or weakened these perspectives. Six [6] studies indicated that predictable funding streams, coordinated procurement processes, and dependable supply chains are essential for the consistent use of digital diagnostics [21,28–30,35,41]. In contexts where financing was institutionalized through government budgets or coordinated donor mechanisms, managers could more effectively plan procurement and maintenance, resulting in fewer service-delivery interruptions for providers. In contrast, unpredictable procurement cycles, fragmented finance, and persistent stockouts undermined diagnostic services, triggering uncertainty and distrust among users and thereby diminishing confidence in the system’s reliability and deterring consistent utilization.

Quality assurance and diagnostic accuracy interacted with policy and financial contexts to influence provider behaviour. Five studies reported that formal quality assurance systems and demonstrated diagnostic accuracy increased clinicians’ confidence in digital diagnostic technologies [17,28,30,31,42]. When quality standards were integrated into regulatory and policy frameworks, providers were more willing to rely on digital outcomes for clinical decision-making, thereby strengthening trust and professional legitimacy.

**Mechanism.** The endorsement of policy and consistent financing indicated a commitment at both the organizational and system levels, hence reducing concerns about the durability and legitimacy of digital diagnostics. This signaling effect influenced the reasoning of managers and providers regarding the intervention: managers emphasized procurement and resource allocation, whereas providers increasingly regarded digital diagnostics as integral to standard, “official” practice rather than as transient or externally imposed initiatives.

**Outcome.** Over time, these processes facilitated lasting integration, enhanced supply continuity, and increased diagnostic uniformity across facilities. Once diagnostics are integrated into existing logistics supply chains and follow standard treatment guidelines, their implementation and sustainability are more likely. For instance, coverage of tests under national health insurance subscriptions further protects patients and households from financial insecurity. In contrast, inadequate policy anchoring and unpredictable procurement conditions created ongoing uncertainty about the availability of tools and institutional support. These conditions undermined trust, interrupted workflows, and led to uneven or selective application of digital diagnostics. Overall, these findings indicate that policy recognition, regulatory clarity, and consistent financing are not only facilitators but also fundamental drivers of the processes by which digital diagnostics are integrated and maintained within African health systems.

#### CMO 5: Data value and health system decision-making.

When digital tools generate actionable data, managers and clinicians recognise their value and reinforce their use by improving system responsiveness.

The capacity of digital diagnostic tools to generate timely, actionable data emerged as an essential means of acknowledging and reinforcing their value for both managers and clinicians. In the literature, digital diagnostics were most effectively sustained and scaled when integrated within data systems that converted test results into operationally useful information, including dashboards, automated reports, or real-time alerts that could guide decision-making across various levels of the health system.

**Context.** In contexts where digital diagnostics were associated with real-time reporting platforms and surveillance systems, stakeholders saw the generated data as credible, timely, and immediately relevant to service delivery needs. These contexts were characterized by the availability of dashboards or automated reporting functions, alongside decision-makers having the technical and organizational capability to analyze and respond to incoming data. Such conditions were particularly common in health systems experiencing significant incidences of febrile illness, when prompt case identification, reporting, and intervention are crucial for both clinical management and public health surveillance [28,30,37].

**Mechanism.** The availability of actionable data influenced stakeholders’ perceptions of the efficacy of digital diagnostics. Managers and clinicians perceived digital outputs not only as documentation of individual test findings but also as indicators that facilitate proactive system management. This included detecting emerging disease patterns, predicting supply shortages, monitoring facility performance, and directing supervisory or supportive measures. As digital data became evidently associated with tangible actions, such as the redistribution of test kits, targeted mentorship, or prompt responses to outbreaks, stakeholders progressively placed their trust in and depended on these systems, thereby reinforcing their integration into routine procedures.

**Outcome.** These mechanisms resulted in various system-level effects. Facilities and districts indicated enhanced supply chain performance, characterized by higher forecasting accuracy and diminished stockouts, as stock management decisions were guided by real-time consumption data. Supervisory processes became more focused, with managers using diagnostic and reporting data to prioritize facilities with significant needs or to address performance deficiencies. The integration of digital diagnostics into surveillance systems improved disease monitoring and response, hence augmenting the health system’s ability to promptly diagnose and address febrile illnesses.

However, the literature also highlighted significant variables. In environments characterized by restricted data literacy, inadequate analytical capacity, or inadequate organizational processes for data response, the expected mechanisms did not materialize. In these situations, digital systems continued to generate data; nevertheless, the lack of interpretive or responsive capabilities led to inaction regarding this data. This led to underutilized dashboards, postponed decision-making, and reduced perceived value among users. Over time, this disconnection diminished engagement with digital diagnostics, illustrating that mere data creation is inadequate; the capacity to convert data into actionable insights is crucial for maintaining and enhancing the utilization of digital diagnostic tools.

## Discussion

This realist review aimed to explain how, and under what circumstances, health systems in Africa integrate digital point-of-care (POC) diagnostics. The review demonstrates that the successful integration of digital point-of-care diagnostics in many African countries depends more on health system readiness to establish trust, perceived usefulness, continuity, institutional legitimacy, and system responsiveness than on the performance of the tests themselves, thereby enabling the routine application of diagnostic data in care and surveillance.

By synthesizing evidence from 17 studies across different countries, we developed an explanatory program theory that shows how specific contextual conditions activate mechanisms that influence health system readiness for digital diagnostic innovations. The findings indicate that digital diagnostics can achieve sustained adoption and systemic impact primarily when integrated into health systems that consistently support their infrastructure, institutionalize their use through policy and financing, and convert diagnostic data into prompt, actionable responses. Together, these findings, along with the prevalence of exploratory and implementation research, indicate the diverse and evolving landscape of digital diagnostics in African health systems. In addition, the pattern of diagnostic studies in the literature suggests that while digital POC diagnostics are widely studied, evidence on newer digital molecular diagnostics remains scarce and underrepresented in the African context.

Overall, the synthesis shows that health systems are ready to integrate digital diagnostic tools when five key system levers align: device usability and workflow compatibility; workforce capacity, trust, and confidence; infrastructure reliability (power, connectivity, and maintenance) and system responsiveness; supportive governance and policy alignment (including financing and supply chain reliability); and digital data systems that deliver value.

When these elements are in place, mechanisms such as perceived usefulness, user confidence, and health system responsiveness lead to outcomes such as sustained adoption, improved diagnostic pathways, and better alignment between treatment decisions and diagnostic evidence. By contrast, weaknesses in any of these areas, especially infrastructure, financing, or supply stability, trigger distrust, user frustration, and non-use, resulting in poor integration.

In line with realist logic, the review emphasizes that integration is not determined solely by the availability of digital tools, but by interactions across system, organizational, and behavioural layers that enable or hinder mechanisms of trust, perceived usefulness, and system responsiveness.

Across studies, usability and acceptability were central mechanisms shaping adoption and integration. Tools that aligned with workflows, required minimal data entry, or provided clinically meaningful outputs triggered confidence among providers and trust among patients, enabling sustained use. In contrast, when technologies increased workload, required complex steps, or failed to meet local diagnostic needs, countermeasures of frustration and avoidance led to discontinued use. This aligns with broader technology adoption literature, which emphasises that perceived ease of use and compatibility with routine practice strongly influence long-term uptake in resource-constrained contexts [23]. Similarly, Ansbro et al. (2015) examining provider perspectives on syphilis POC implementation in Zambian antenatal clinics, found that acceptability depended on perceived clinical relevance and fit with existing consultation workflows, reflecting the user frustration and avoidance pattern identified here [43].

A complementary NASSS-based mixed methods systematic review of RDT implementation determinants in sub-Saharan Africa supports these findings, highlighting limited integration into existing clinical workflows and misalignment between test results and clinical judgment as significant barriers to sustained uptake across 33 countries [44]. This review also highlighted that the single focus of most RDTs diminishes their effectiveness in situations where patients often exhibited undifferentiated fever or co-existing conditions. This design limitation interacts with and exacerbates the systemic readiness challenges identified in this realist review, which may also contribute to the misalignment between test results and clinical judgment observed in both bodies of evidence.

Workforce capacity was a critical contextual lever that activated competence and trust mechanisms. Training, mentorship, and supportive supervision strengthened providers’ confidence in digital test results and promoted adherence to diagnostic protocols. However, high workloads and ongoing staffing shortages, especially in low-resource primary care centers, undermined these mechanisms. Even well-designed tools were underutilized when providers felt overwhelmed or unable to reorganize workflows around digital processes, demonstrating that structural workforce constraints can overwhelm otherwise enabling mechanisms. Baldeh et al. (2026) reinforce that increased health worker workload is a consistently reported system limitation to sustained adoption of RDTs in SSA contexts with evidence indicating that overburdened health providers often deprioritize diagnostic testing even when they acknowledge its importance. Examples from digital decision-support contexts provide supporting evidence. Ngassa Piotie et al. (2021) showed that structured training for the TIP insulin management platform in South Africa led to significant improvements in nurses’ competence and independent clinical decision-making, which reflects a pattern closely aligned with the competence-driven confidence mechanism described here [45].

Another structural challenge is high staff turnover, which the reviewed evidence identifies as a contextual feature of many PHC settings (see CMO 2, Context). In contrast to staffing shortages, which reduce capacity at specific points in time, staff turnover undermines institutional memory and the accumulated competencies required to sustain the use of digital diagnostic tools. Furthermore, turnover disrupts training continuity, requires constant reinvestment in capacity building and poses risks to trust in digital tools that develop over time with consistent use. None of the 17 studies included in the review explicitly examined staff turnover as an independent variable, however, its interaction with training and supervision mechanisms was clear in the contextual description of multiple studies. Addressing turnover in the design of strategies for digital diagnostics deployment requires further attention in future implementation research.

Infrastructure and connectivity were both enabling and limiting factors. Reliable power, device maintenance, and connectivity enabled real-time data transmission, triggering system responses such as improved stock management, timely feedback, and enhanced surveillance. Conversely, infrastructure weaknesses caused disruptions, resulting in broken data flows, device downtime, and a fallback to paper-based systems. This pattern mirrors the broader realities of digital health in Africa, where inconsistent infrastructure determines whether digital systems can achieve their intended outcomes. For instance, findings from a district hospital in Malawi highlight how unstable electricity and a lack of device maintenance were the main drivers behind the discontinuation of POC ultrasound use. Providers reported a gradual decline in trust in the technology [46], which aligns directly with the adverse pathway of CMO 3. This cross-country, cross-condition pattern indicates that unreliable infrastructure functions as a suppressive mechanism, irrespective of the diagnostic tool or clinical condition.

Governance, policy, and financing are key contextual factors influencing readiness at higher system levels. Incorporating digital diagnostics into national policies and guidelines legitimized new interventions. Additionally, predictable financing and reliable procurement processes ensured consistent access to and use of these diagnostics. In contrast, irregular procurement cycles and stockouts can undermine provider trust and disrupt service delivery. The governance mechanism identified in this review is further corroborated by broader literature on POC implementation. In Ghana, fragmented financing and unreliable procurement were primary governance limitations to the integration of POC diagnostics, regardless of providers’ willingness to use the devices [47]. Similarly, a Zambian study found that even with policy endorsement of syphilis POC testing, it was not sufficient to ensure sustained use by health workers in the context of unreliable procurement systems and stockouts, mirroring uncertainty and distrust configurations described in this review [43]. These studies confirm that the institutional legitimacy mechanism is not exclusive to digital POC diagnostics but is a characteristic health-systems response to any POC technology in the absence of robust policy and supply-chain infrastructure.

Furthermore, quality assurance systems that demonstrate diagnostic accuracy help reinforce trust among both providers and patients. This illustrates the intersection of technical performance and governance in sustaining the use of these tools.

### Contribution to existing knowledge

This evidence synthesis highlights that health system readiness to integrate digital POC diagnostics is not determined by the presence of discrete components but rather emerges from the alignment of system conditions that enable or suppress key mechanisms. Five key health system levers, namely: (1) device usability and acceptability, (2) workforce capacity, trust and confidence, (3) stable infrastructure and system responsiveness, (4) supportive governance and policy alignment and financing arrangements and (5) Data value and health systems decision-making, were shown to matter not as independent determinants, but insofar as they shaped whether mechanisms such as user confidence, perceived clinical usefulness, professional legitimacy, and system responsiveness were activated as shown in Fig 2. When these conditions aligned, digital POC diagnostics were more likely to be trusted, acted upon and embedded into routine practice, resulting in sustained adoption and improved diagnostic pathways. These findings emphasize that the usefulness of digital diagnostics is achieved through the integration of generated data into adaptive organizational and systems processes. When actionable data are integrated with the capacity for decision-making and established response pathways, they build confidence, value, and reinforcement mechanisms that promote sustainable adoption and system improvement.

Conversely, weaknesses in any of these areas, particularly infrastructure reliability, financing, or supply stability, disrupted the activation of these mechanisms, instead generating distrust, frustration, and disengagement that undermined effective integration. These findings help explain why technically sound diagnostic innovations often fail to achieve sustained use in resource-constrained settings.

By applying a realist lens, this review extends prior work on POC and digital diagnostics in Africa by moving beyond descriptive accounts of health system facilitators and barriers [18,21] to offer explanatory insights into how, why and under what conditions digital diagnostics are integrated, fully or partially, or abandoned. In doing so, the study reframes digital diagnostics as socio-technical and system-wide interventions whose uptake depends not only on technological performance but on institutional legitimacy, organizational capacity and stakeholder trust. This theory-informed contribution provides a transferable framework to guide future research, policy and implementation strategies for digital diagnostics in African health systems.

### Strengths and limitations

A key strength of this review lies in its use of a realist approach, which enabled the synthesis of diverse qualitative, quantitative and mixed-methods evidence to generate transferable explanatory programme theories. By focusing on how and why digital POC diagnostics are integrated or fail to be integrated into health systems across different African contexts, the review moves beyond descriptive accounts of barriers and facilitators to develop context-sensitive CMO configurations that can inform policy, implementation and innovation in health systems facing comparable structural constraints. The refined program theory aligns with and extends established social science and implementation science frameworks and theories, such as the Technology Acceptance Model (TAM) and the Unified Theory of Acceptance and Use of Technology (UTAUT) [48,49]. While these models emphasize individual-level determinants of adoption, this realist review adds explanatory value by foregrounding institutional legitimacy, organizational conditions, and system-level alignment, and by explicitly accounting for the mechanisms underlying non-adoption, partial integration, and abandonment. In doing so, it shifts analytical attention away from the intrinsic attributes of diagnostic technologies alone and toward the diagnostic ecosystem and broader health system within which diagnostics are embedded.

The evidence base, although conceptually rich, has several limitations. A substantial proportion of included studies were conducted in South Africa (5/17; 29%), which may have skewed findings toward settings with relatively stronger infrastructure, governance capacity and digital maturity compared to other African contexts. This concentration limits the review’s generalizability, although the aim of the realist review was to support theoretical transferability rather than representativeness. In addition, most studies focused on routine POC diagnostics rather than on advanced digital molecular diagnostic technologies, which remain underexplored in real-world African health systems. Financing models and long-term sustainability were not thoroughly explored, with few studies incorporating robust economic or health financing evaluations.

Despite these limitations, the review offers valuable insights into the conditions under which digital POC diagnostics can be successfully integrated into health systems and highlights the multiple interacting levels of readiness that shape implementation outcomes. The findings offer a coherent and testable framework, while also pointing to important gaps in the existing literature.

### Future research

Several priority areas warrant further investigation, based on the findings of this review. Firstly, there is a need for prospective empirical testing of the refined CMO configurations and pathways across diverse African health system contexts, to assess their explanatory power and further refine the programme theories. Such studies would strengthen understandings of how institutional, organisational and professional contexts interact to shape readiness and sustainability.

Secondly, longitudinal research is needed to frame and examine how diagnostic ecosystems, encompassing production, supply chains, maintenance, quality assurance, supervision, and data governance, affect diagnostic fidelity, provider trust, and long-term integration beyond pilot or donor-funded projects. Attention to temporal findings is particularly important because readiness is conditional, dynamic and reversible.

Finally, more emphasis is needed on equity, affordability, and health financing. Future studies should assess whether integrating digital diagnostics narrows or widens disparities in access and outcomes, particularly among underserved rural and marginalized populations, and examine how financing models influence sustainability and scale. Addressing these gaps will be essential to ensuring that digital diagnostics contribute to equitable and resilient health systems and improved health outcomes.

## Conclusion

This realist review demonstrates that health system readiness to integrate digital POC diagnostics in Africa is not a fixed precondition but an emergent and reversible outcome. Readiness arises when institutional, organizational, and technological conditions align to activate mechanisms of trust, confidence, professional legitimacy, and responsiveness to diagnostic information. Health system levers, including (i) usability and acceptability; (ii) workforce capacity; (iii) infrastructure and connectivity; (iv) governance, policy alignment, and financing; and (v) data value and decision-making systems, matter not as independent determinants but as interlinked conditions that enable or constrain the activation of these mechanisms. Where the mechanisms are triggered, they lead to sustained diagnostic adoption and strengthened diagnostic pathways; where they are suppressed, mechanisms of frustration and disengagement undermine diagnostic integration, regardless of technical performance.

By highlighting the contextual conditions that shape the activation of mechanisms that lead to varying outcomes, this review advances a theory-informed understanding of health system readiness for integrating digital POC diagnostics. The refined programme theory offers a practical framework to support policymakers, implementers, and funders in tailoring digital diagnostic strategies to local health system readiness. The findings underscore that successful integration of digital diagnostics in African health systems will depend not only on technological innovations but also on sustained investment in, and strengthening of institutional legitimacy, organisational capacity, and governance arrangements that enable diagnostic tools and information to be trusted, acted upon, and maintained over time.

## Supporting information

S1 Text Search terms. (DOCX)

S1 File Covidence extraction matrix. (CSV)

S2 File CMO synthesis table and thematic categorization of literature into Context-Mechanism-Outcome configurations (CMO). (XLSX)

S1 Acknowledgements NIHR Global Health Research Group on Digital Diagnostics for African Health Systems (DIDA). (XLSX)
