# Supplementary material for: Health system readiness for digital point-of-care diagnostics for febrile illness across Africa: A realist review
Source: PLOS Digit Health. 2026 Nov 6;5(11):e0001820. doi: 10.1371/journal.pdig.0001820 (PMC13742922; doi:10.1371/journal.pdig.0001820)
Supplement: Supplementary file 1 [file pdig.0001820.s001.docx]

Search terms

Pubmed (January 1, 2005 – August 20, 2025)

(((("Point-of-Care Diagnostic Testing" OR "Point-of-Care Diagnostic Test" OR "Rapid Diagnostic" OR "Digital health" OR "Digital Diagnostics" OR "lab-on-a-chip") AND ("Health Systems" OR "Healthcare Integration" OR "Health Service Delivery" OR "Health System Sustainability" OR "Healthcare Infrastructure" OR health*)) AND ("Africa" OR "Sub-Saharan Africa")) AND ("Integrat*" OR "Implement*" OR "Sustain*" OR "Adopt*" OR "Scale-up"))

CINAHL (January 1, 2005 – August 20, 2025)

(((MH "Point-of-Care Systems+" OR ("lab-on-a-chip" OR "Point-of-Care Diagnostic Test" OR "digital diagnostic" OR "Point-of-Care Diagnostic Testing" OR "Digital Diagnostics"))) AND ((MH "Health Services Administration+" OR ("Health Systems" OR "Healthcare Integration" OR "Health Service Delivery"))) AND ((MH "Africa+" OR ("sub-saharan africa" OR "sub-saharan" OR africa))) AND (("Integrat*" OR "Readiness" OR "Implement*" OR "Adopt*" OR "Scale-up")) NOT (("review of literature" OR "meta-analysis" OR "systematic review" OR "scoping review"))
