# Supplementary material for: Health system readiness for digital point-of-care diagnostics for febrile illness across Africa: A realist review
Source: PLOS Digit Health. 2026 Nov 6;5(11):e0001820. doi: 10.1371/journal.pdig.0001820 (PMC13742922; doi:10.1371/journal.pdig.0001820)
Supplement: Supplementary file 2 [file pdig.0001820.s006.docx]

**Response to Reviewers**

We sincerely thank the editor and both reviewers for their constructive and detailed comments, which have strengthened the manuscript. We provide point-by-point responses in the table below as requested. All line numbers refer to the revised manuscript with tracked changes.

We hope that the above responses and revisions adequately address the concerns raised. We remain available to provide any further clarification the editor or reviewers may require.

Yours sincerely,

Shola Molemodile Dele-Olowu (on behalf of all co-authors)

|  | **Comments** | **Response** |
| --- | --- | --- |
|  | **Reviewer 1** |  |
| 1 | The manuscript is relevant to the publisher's scope. | We thank the reviewer for this positive feedback with regards to the fit between the manuscript and the journal. |
| 2 | However, the word 'variation' when referencing current POC diagnostics is ambiguous and may be perceived negatively. The authors are recommended to define what is meant by variation in this context. Is there evidence that the same person received two different results when utilising the current POCs. | We thank the reviewer for this important observation. The term 'variation' was used throughout to describe inter-setting differences in how existing POC diagnostics (such as malaria RDTs) have been introduced, adopted, and scaled-up across African health systems, not intra-patient diagnostic variability or test performance inconsistency. We recognise that the original phrasing was ambiguous and could be misread as implying unreliability of the tests themselves.  We have revised all relevant uses of the term 'variation' in the Introduction and Discussion to explicitly describe 'variation in implementation and scale-up across health systems and settings'. Specifically, the sentence in lines 87 - 89 now reads: '...while these initiatives expanded diagnostic availability, they also highlighted substantial variation in how new diagnostics were introduced and scaled up across different health system contexts.' This makes clear that we are defining variation as a feature of implementation pathways, and not as diagnostic test (in)accuracy. |
| 3 | The numbering of references does not correspond to the number of references listed. Furthermore, the referencing is not consistent between text and reference listing. This is major as it implies Authors mentioned to have used references which do not exist. | We thank the reviewer for spotting this error which we have corrected by reformatting the reference list in Vancouver style and restructured to be numbered in order of first appearance in the text, ensuring all in-text citation numbers correspond directly to the reference list  During this careful cross-checking process, two additional errors were spotted and corrected:  **Two duplicate entries removed:** Greenhalgh et al. (2017) was listed twice (as '2017a' and '2017b') but both entries cite the identical paper (JMIR, 19(11), e367, doi:10.2196/jmir.8775). Similarly, Karemere et al. (2025) was listed twice (as '2025a' and '2025b') but both entries cite the identical paper (Am J Trop Med Hyg, 112(1_Suppl), 10–16). One entry from each duplicate pair has been removed.  **Missing references:** The submitted reference list contained 42 entries (40 unique after removing duplicates), but the in-text citations span numbers 1–50 (49 unique citation numbers, with no citation #48 used). This means approximately 9 references cited in the text were absent from the reference list. These have all been updated |
|  | **Reviewer 2** |  |
| 4 | This manuscript describes a realist review to examine how, why, and under what circumstances digital POC diagnostics for febrile illness are integrated into African health systems. Based on data from twenty-one articles from sixteen African countries, the authors found that the integration of digital point-of-care diagnostics into African health systems depends more on the health system’s readiness to promote trust, continuity, and data-driven action than on the performance of the tests themselves. The most frequently cited characteristics influencing integration were usability and acceptability; ten studies reported that perceived ease of use, workflow compatibility, and clinical relevance and usefulness shaped uptake. The results are categorized into a Context-Mechanism-Output (CMO) map under the themes of workforce, infrastructure and connectivity, governance, policy and finance as shown in S6 Table 3. This is a well-written review on an important and timely topic. The aim of the review is clearly stated, methods well-described and the results provide very useful information on how digital POC diagnostics can be successfully implemented in Africa. However, there are several areas of concern that the authors need to address. | We thank Reviewer #2 for summarizing the manuscript and for your positive feedback on the importance and timeliness of the topic. We are pleased that the reviewer finds the aim clearly stated, the methods well-described and the results useful. Specific comments are addressed directly below. |
| 5 | The title 'Health system readiness for digital point-of-care diagnostics for febrile illness across Africa' is misleading as at least half the articles did not involve POC testing. POC testing was mentioned in article 4 from Ghana but not used in association with febrile illness or any digital technology. Articles 6, 9, 12 are about ultrasound, syphilis screening and insulin management respectively, and not febrile illness. Article 9 described the implementation of POC syphilis tests but did not involve any digital technology at all. Perhaps the short title 'Health system readiness for digital point-of-care diagnostics in Africa' is more appropriate. | We thank the reviewer for this important and valid observation, and we have made significant changes to address it in two ways.   1. We have removed four flagged articles:   **Article 4:** Ansu-Mensah et al. (2024), Ghana - POC barriers without specific digital technology.  **Article 6:** Limani et al. (2021), Malawi - POC ultrasound (POCUS), neither molecular/connected nor febrile illness specific.  **Article 9:** Ansbro et al. (2015), Zambia - syphilis RST without digital technology.  **Article 12 :** Ngassa Piotie et al. (2021), South Africa - digital intervention for insulin management, not febrile illness.   2. We retained other studies that address digital POC implementation more broadly because they provide essential mechanistic evidence for the programme theory following RAMESES realist review methodology. In this case, the studies are retained because they produce transferable evidence regarding health system mechanisms i.e. usability, workforce competence, infrastructure reliability, governance, and data value, that are directly applicable to febrile illness digital point-of-care contexts. This is clearly described and justified in the methods   With these changes, we respectfully retain ‘for febrile illness’ in the title as the changes resolve the reviewer’s concern (five studies addressing febrile illness diagnostics directly (Ebola RDT; automated malaria RDT reader; pneumonia/ARI screening; AI-enabled malaria diagnostics; REASSURED digital-linked POC) and all 17 addressing implementation mechanisms in the same contexts where febrile illness diagnostics are deployed). Retaining ‘febrile illness’ also differentiates this review from the growing body of evidence of digital POC in Africa more broadly and highlights the specific contribution for the evidence gap concerning febrile illnesses. |
| 6 | The references in the text are numbered and yet under References, the articles are in alphabetical order. So, it is impossible to look up details of studies using the citations in the text. | We thank the reviewer for spotting this error which, as noted above, we have corrected by reformatting the reference list in Vancouver style has been restructured to use sequential numbering in order of appearance, ensuring all in-text citations correspond directly to the reference list  During this careful cross-checking process, two additional errors were spotted and corrected:  **Two duplicate entries removed:** Greenhalgh et al. (2017) was listed twice (as '2017a' and '2017b') but both entries cite the identical paper (JMIR, 19(11), e367, doi:10.2196/jmir.8775). Similarly, Karemere et al. (2025) was listed twice (as '2025a' and '2025b') but both entries cite the identical paper (Am J Trop Med Hyg, 112(1_Suppl), 10–16). One entry from each duplicate pair has been removed.  **Missing references:** The submitted reference list contained 42 entries (40 unique after removing duplicates), but the in-text citations span numbers 1–50 (49 unique citation numbers, with no citation #48 used). This means approximately 9 references cited in the text were absent from the reference list. These have all been updated accordingly |
| 7 | S3 Table 2. Characteristics of articles examined for the study did not include any citations. So, it was impossible to look up the articles/studies mentioned. It also did not contain any information on the POC tests used. Any mention of POC testing is only from the title of the study. | We thank the reviewer for identifying these gaps. We have revised Table 2, which has now also been moved into the main manuscript, with two new columns added:  **Column: Reference.** Each row of Table 2 now includes the in-text citation corresponding to each included study, enabling direct cross-referencing with the updated reference list.  **Column: POC Technology / Diagnostic Tool.** Each row now specifies the diagnostic technology included in the review. Based on the manuscript text and reference list, the technologies included across the 17 original studies encompass: malaria rapid diagnostic tests (RDTs); AI-enabled/connected RDT readers (e.g. ConnDx); automated malaria RDT readers; digital reporting systems; mHealth platforms; digital POC platforms for infectious diseases; digital molecular diagnostics (lab-on-chip); and broader digital health systems. |
| 8 | Workforce capacity was a critical contextual lever. The authors mentioned high workloads and staff shortages as challenges. High staff turnover has been noted in many studies on the implementation of POC testing. Did the authors come across this challenge in their review? | We thank the reviewer for raising this important point. Although high staff turnover was briefly acknowledged in the manuscript as a contextual factor in the CMO 2 section of the Methods/Results, we agree that this was not adequately carried forward into the mechanistic analysis or Discussion.  While in the previous version, staffing shortages and heavy workloads were analysed as mechanisms undermining training benefits, we have now strengthened the Discussion section by including the distinct challenge of staff turnover, which disrupts institutional memory, interrupts competence-building, and erodes the trust and familiarity that providers develop with digital tools over time (please see lines 480-489). |
| 9 | S5 Figure 2 is an important figure which should be in the main text and not as supplementary material. | We agree with this recommendation. Figure 2, which illustrates the refined programme theory developed through this realist review, showing how five key health system levers interact with mechanisms to produce or undermine readiness for digital POC diagnostic integration, is the primary analytical output of the paper. Figure 2 has therefore been moved from the supplementary files section into the main manuscript. It now appears in the Results section, immediately after the description of CMO 5 (following line 412). The figure file has been reprocessed through the PLOS NAAS tool and resubmitted as a main figure file. |
