# Supplementary material for: Health system readiness for digital point-of-care diagnostics for febrile illness across Africa: A realist review
Source: PLOS Digit Health. 2026 Nov 6;5(11):e0001820. doi: 10.1371/journal.pdig.0001820 (PMC13742922; doi:10.1371/journal.pdig.0001820)
Supplement: Peer review history

# Peer review: Decision Letter 0

**DOI:** 10.1371/journal.pdig.0001820.r001

**Authors:** Thomas Kisimbi, Shuduo Zhou

1 Jun 2026

Response to Reviewers Revised Manuscript with Track Changes Manuscript **Journal Requirements:**

1. Please amend your detailed Financial Disclosure statement. This is published with the article. It must therefore be completed in full sentences and contain the exact wording you wish to be published.

a. State the initials, alongside each funding source, of each author to receive each grant.

b. State what role the funders took in the study. If the funders had no role in your study, please state: “The funders had no role in study design, data collection and analysis, decision to publish, or preparation of the manuscript.”

c. If any authors received a salary from any of your funders, please state which authors and which funders.

2. We have noticed that you have cited Table 1 and 2 in the manuscript file but there are no corresponding tables in the manuscript. Please amend your manuscript to include this table, noting that tables should not be uploaded as individual files.

3. We note that your Data Availability Statement is currently as follows: "All relevant data are contained within the manuscript and its Supporting Information files. The data used in this study consist of published literature identified through systematic database searches and purposive grey literature searches. Extraction matrices and context–mechanism–outcome (CMO) synthesis tables supporting the findings of this review are available from the corresponding author upon reasonable request."

Please confirm at this time whether or not your submission contains all raw data required to replicate the results of your study. Authors must share the “minimal data set” for their submission. PLOS defines the minimal data set to consist of the data required to replicate all study findings reported in the article, as well as related metadata and methods (https://journals.plos.org/plosone/s/data-availability#loc-minimal-data-set-definition).

For example, authors should submit the following data:

- The values behind the means, standard deviations and other measures reported;

- The values used to build graphs;

- The points extracted from images for analysis.

Authors do not need to submit their entire data set if only a portion of the data was used in the reported study.

If your submission does not contain these data, please either upload them as Supporting Information files or deposit them to a stable, public repository and provide us with the relevant URLs, DOIs, or accession numbers. For a list of recommended repositories, please see https://journals.plos.org/plosone/s/recommended-repositories.

If there are ethical or legal restrictions on sharing a de-identified data set, please explain them in detail (e.g., data contain potentially sensitive information, data are owned by a third-party organization, etc.) and who has imposed them (e.g., an ethics committee). Please also provide contact information for a data access committee, ethics committee, or other institutional body to which data requests may be sent. If data are owned by a third party, please indicate how others may request data access.

**Additional Editor Comments (if provided):** **Reviewers' Comments:**

**Comments to the Author**

1. Does this manuscript meet PLOS Digital Health’s publication criteria?

Reviewer #1: Yes

Reviewer #2: Yes

**********

2. Has the statistical analysis been performed appropriately and rigorously?

Reviewer #1: N/A

Reviewer #2: N/A

**********

3. Have the authors made all data underlying the findings in their manuscript fully available (please refer to the Data Availability Statement at the start of the manuscript PDF file)?

The PLOS Data policy

Reviewer #1: Yes

Reviewer #2: Yes

**********

4. Is the manuscript presented in an intelligible fashion and written in standard English?

Reviewer #1: Yes

Reviewer #2: Yes

**********

Reviewer #1: The manuscript is relevant to the publisher’s scope. however the word variation when referencing to current POC is ambiguous and may be perceived negatively. The authors are recommended to define what is meant by variation in this context. Is there evidence that the same person received two different results when utilising the current POCs.

The numbering of references does not correspond to the number of references listed. Furthermore, the referencing is not consistent between text and reference listing. This is major as it implies Authors mentioned to have used references which do not exist.

Reviewer #2: This manuscript describes a realist review to examine how, why, and under what circumstances digital POC diagnostics for febrile illness are integrated into African health systems. Based on data from twenty-one articles from sixteen African countries, the authors found that the integration of digital point-of-care diagnostics into African health systems depends more on the health system’s readiness to promote trust, continuity, and data-driven action than on the performance of the tests themselves.

The most frequently cited characteristics influencing integration were usability and acceptability; ten studies reported that perceived ease of use, workflow compatibility, and clinical relevance and usefulness shaped uptake. The results are categorized into a Context-Mechanism-Output (CMO) map under the themes of workforce, infrastructure and connectivity, governance, policy and finance as shown in S6 Table 3.

This is a well-written review on an important and timely topic. The aim of the review is clearly stated, methods well-described and the results provide very useful information on how digital POC diagnostics can be successfully implemented in Africa. However, there are several areas of concern that the authors need to address. Specifically,

1. The title, “Health system readiness for digital point-of-care diagnostics for febrile illness across Africa: a realist review” is misleading as at least half the articles did not involve POC testing. POC testing was mentioned in article 4 from Ghana but not used in association with febrile illness or any digital technology. Articles 6, 9, 12 are about ultrasound, syphilis screening and insulin management respectively, and not febrile illness. Article 9 described the implementation of POC syphilis tests but did not involve any digital technology at all. Perhaps the short title, “Health system readiness for digital point-of-care diagnostics in Africa” is more appropriate.

2. The references in the text are numbered and yet under References, the articles are in alphabetical order. So, it is impossible to look up details of studies using the citations in the text.

3. S3 Table 2. Characteristics of articles examined for the study did not include any citations. So, it was impossible to look up the articles/studies mentioned. It also did not contain any information on the POC tests used. Any mention of POC testing is only from the title of the study.

4. Workforce capacity was a critical contextual lever. The authors mentioned high workloads and staff shortages as challenges. High staff turnover has been noted in many studies on the implementation of POC testing. Did the authors come across this challenge in their review?

5. S5 Figure 2 is an important figure which should be in the main text and not as supplementary material.

**********

what does this mean?). If published, this will include your full peer review and any attached files.

**Do you want your identity to be public for this peer review?**  If you choose “no”, your identity will remain anonymous but your review may still be made public.

For information about this choice, including consent withdrawal, please see our Privacy Policy

Reviewer #1: **Yes:**  Boitumelo Moetlhoa

Reviewer #2: No

**********

**Figure resubmission:**

**Reproducibility:**

To enhance the reproducibility of your results, we recommend that authors of applicable studies deposit laboratory protocols in protocols.io, where a protocol can be assigned its own identifier (DOI) such that it can be cited independently in the future. Additionally, PLOS ONE offers an option to publish peer-reviewed clinical study protocols. Read more information on sharing protocols at https://plos.org/protocols?utm_medium=editorial-email&utm_source=authorletters&utm_campaign=protocols

# Author comment: Author response to Decision Letter 1

**DOI:** 10.1371/journal.pdig.0001820.r002

25 Jun 2026

Attachment Submitted filename: Response to Reviewers_PLOSDH_22.6.26.docx

# Peer review: Decision Letter 1

**DOI:** 10.1371/journal.pdig.0001820.r003

**Authors:** Shuduo Zhou

15 Oct 2026

Health system readiness for digital point-of-care diagnostics for febrile illness across Africa: a realist review

PDIG-D-26-00403R1

Dear Dr Dele-Olowu,

We are pleased to inform you that your manuscript 'Health system readiness for digital point-of-care diagnostics for febrile illness across Africa: a realist review' has been provisionally accepted for publication in PLOS Digital Health.

Before your manuscript can be formally accepted you will need to complete some formatting changes, which you will receive in a follow-up email from a member of our team.

Please note that your manuscript will not be scheduled for publication until you have made the required changes, so a swift response is appreciated.

IMPORTANT: The editorial review process is now complete. PLOS will only permit corrections to spelling, formatting or significant scientific errors from this point onwards. Requests for major changes, or any which affect the scientific understanding of your work, will cause delays to the publication date of your manuscript.

If your institution or institutions have a press office, please notify them about your upcoming paper to help maximize its impact. If they'll be preparing press materials, please inform our press team as soon as possible -- no later than 48 hours after receiving the formal acceptance. Your manuscript will remain under strict press embargo until 2 pm Eastern Time on the date of publication. For more information, please contact digitalhealth@plos.org.

Thank you again for supporting Open Access publishing; we are looking forward to publishing your work in PLOS Digital Health.

Best regards,

Shuduo Zhou, Ph.D.

Section Editor

PLOS Digital Health

***********************************************************

**Additional Editor Comments (if provided):**

**Reviewer Comments (if any, and for reference):**

Reviewer’s Responses to Questions

**Comments to the Author**

Reviewer #2: All comments have been addressed

**********

publication criteria?

Reviewer #2: Yes

**********

3. Has the statistical analysis been performed appropriately and rigorously?

Reviewer #2: N/A

**********

4. Have the authors made all data underlying the findings in their manuscript fully available (please refer to the Data Availability Statement at the start of the manuscript PDF file)?

The PLOS Data policy

Reviewer #2: Yes

**********

5. Is the manuscript presented in an intelligible fashion and written in standard English?

Reviewer #2: Yes

**********

Reviewer #2: The authors have addressed all the reviewers' concerns.

**********

what does this mean?). If published, this will include your full peer review and any attached files.

**Do you want your identity to be public for this peer review?**  If you choose “no”, your identity will remain anonymous but your review may still be made public.

For information about this choice, including consent withdrawal, please see our Privacy Policy

Reviewer #2: **Yes:**  Rosanna W Peeling

**********
